# Provincial Medical and Surgical Association

**Published:** 1840-07

**Authors:** 


### PROVINCIAL MEDICAL AND SURGICAL ASSOCIATION.

THE Eighth Anniversary Meeting of this Association will be held at Southampton, on Wednesday, the 22d, and Thursday, the 23d of July next. We understand that the following will be the general course of the proceedings:

The first general meeting of the association will be held at the Audit House, on Wednesday, July 22, and the president, Dr. Jeffreys, of Liverpool, will resign the chair at half-past two, p. m. to Dr. Steed, the president-elect, who will then address the meeting, after which the report of the council will be read by the secretary, and other necessary business will be transacted. On this occasion the Thackeray prize for the best essay "On the Causes and Mode of Propagation of Fever" will be adjudicated.

On Wednesday evening, at eight p. m., the members will again assemble at the same place, when Mr. Dodd, of Chichester, will read a report on the progress of Surgery.

On Thursday morning, the 23d, at nine o'clock, the members of the association and their friends will breakfast together at the Royal Victoria Archery Rooms.

At twelve o'clock the same day a general meeting of the members will again be held at the Audit House, and the retrospective address will be delivered by Dr. Scott, of Liverpool, and the reports of committees will be received.

On Thursday evening, at six o'clock, the members and their friends will dine together at the Royal Victoria Archery Rooms, and at nine o'clock the same evening a conversational meeting will be held.

This is expected to be one of the most numerously attended and interesting meetings yet held by the Association; to the success of which, no doubt, the facilities of access offered by the railway, now open between London and Southampton, will not a little contribute. Every succeeding year, since the formation of this Association, has tended more fully to demonstrate its utility, and added to the number of its members, who are now nearly TWELVE HUNDRED, comprising many of the most eminent men in the profession, in all parts of the kingdom. The union of so numerous a body of practitioners cannot fail to afford facilities for the advancement of medical science, which could not otherwise be obtained, as well as to exercise a very powerful influence on all public and legislative measures, affecting the interests of the profession; while the anniversary meetings, by bringing into friendly intercourse the widely separated members, eminently conduce to diffuse a general spirit of kindness and courtesy throughout its various branches.

The eighth volume of the Transactions of the Association has just made its appearance; but, we regret to say, at so late a period of the quarter, as to preclude our giving any detailed account of its contents. We cannot, however, let the opportunity pass of calling the attention of the profession, in this and other countries, to the most valuable memoir of Mr. Ceeley contained in it, entitled "Observations on the Variolæ Vaccinæ as they occasionally appear in the Vale of Aylesbury, with an account of some recent experiments on the Vaccination, Retrovaccination, and Variolation of Cows." This memoir extends to 150 pages, and is illustrated by thirty-five beautifully coloured plates. We entirely concur in the following judgment pronounced on this paper by the council in the preface to the volume: "Several questions of the utmost interest to the welfare of the community generally, as well as the medical science, will be found to be most ably investigated. The variolation of the cow has been satisfactorily accomplished, with the effect of thereby generating the vaccine; the characters of the vaccine in the cow, with several of its irregularities, are carefully described and figured, and some of the most nearly allied spurious forms pointed out, by attention to which many of the difficulties attendant upon a recurrence to the cow for renewed supplies of the vaccine may be obviated. Considerable light has also been thrown upon the value of the practice of retro-vaccination as a means of renewing and restoring the properties of lymph presumed to be deteriorated or otherwise altered by repeated transmissions through the human body; while the paper altogether must form a standard of reference for those who are engaged in the endeavour to extend and perfect the vaccine as a means of protecting millions from the fatal ravages of the smallpox."

